# hoDCA: higher order direct-coupling analysis

**DOI:** 10.1186/s12859-018-2583-6

**Published:** 2018-12-29

**Authors:** Michael Schmidt, Kay Hamacher

**Affiliations:** 1 Department of Physics, TU Darmstadt, Karolinenpl. 5, Darmstadt, 64287 Germany; 2 Department of Biology, TU Darmstadt, Schnittspahnstr. 10, Darmstadt, 64287 Germany; 3 Department of Computer Science, TU Darmstadt, Karolinenpl. 5, Darmstadt, 64287 Germany

**Keywords:** Contact prediction, Proteins, DCA

## Abstract

**Background:**

Direct-coupling analysis (DCA) is a method for protein contact prediction from sequence information alone. Its underlying principle is parameter estimation for a Hamiltonian interaction function stemming from a maximum entropy model with one- and two-point interactions. Vastly growing sequence databases enable the construction of large multiple sequence alignments (MSA). Thus, enough data exists to include higher order terms, such as three-body correlations.

**Results:**

We present an implementation of hoDCA, which is an extension of DCA by including three-body interactions into the inverse Ising problem posed by parameter estimation. In a previous study, these three-body-interactions improved contact prediction accuracy for the PSICOV benchmark dataset. Our implementation can be executed in parallel, which results in fast runtimes and makes it suitable for large-scale application.

**Conclusion:**

Our hoDCA software allows improved contact prediction using the Julia language, leveraging power of multi-core machines in an automated fashion.

## Background

Thanks to rapidly growing sequence databases, the prediction of protein contacts from sequence information has become an promising route for computational structural biophysics [1–4]. The so called direct-coupling analysis (DCA) uses a multiple sequence alignment (MSA) to predict residue contacts in a maximum entropy approach. Its high accuracy was shown in various studies [5–11] and also made it suitable for protein structure prediction software [12–14].

The DCA approach leads to a Potts model with probability for a sequence $\vec {\sigma } =\left (\sigma _{1},\dots,\sigma _{N}\right)$ given as $P(\vec {\sigma })=\text {exp}\left [- H(\vec {\sigma }) \right ]/Z$, with Hamiltonian $H(\vec {\sigma }) = -{\sum \nolimits }_{i}^{N} h_{i}\left (\sigma _{i}\right) - {\sum \nolimits }_{1 \leq i< j \leq N} J_{ij}\left (\sigma _{i},\sigma _{j}\right)$ consisting of local fields and two-body interactions and *N* being the length of the sequences. $Z={\sum \nolimits }_{\vec {\sigma }\in \mathcal {A}^{N}} P(\vec {\sigma })$ is the partition function as the sum over all sequences where each position is chosen from the alphabet $\mathcal {A}$. After estimation of parameters {*h*_*i*_,*J*_*ij*_} from empirical sequences $\vec {\sigma }^{(b)}$, a contact prediction score for residue *i* and *j* can be obtained by taking the *l*_2_-norm ∥*J*_*ij*_∥_2_. In a recent study [15], an improved prediction accuracy was shown by incorporating three-body interactions *V*_*ijk*_(*σ*_*i*_,*σ*_*j*_,*σ*_*k*_) into *H*, obtaining a three-body Hamiltonian

$$\begin{array}{@{}rcl@{}} H^{(3)}(\vec{\sigma}) &=& -\sum\limits_{i}^{N} h_{i}\left(\sigma_{i}\right) \\ && - \sum\limits_{1 \leq i< j \leq N} J_{ij}\left(\sigma_{i},\sigma_{j}\right)\\ && - \sum\limits_{1 \leq i< j< k \leq N} V_{ijk}\left(\sigma_{i},\sigma_{j},\sigma_{k}\right). \end{array} $$

Here, we present an implementation of this method, which we call hoDCA.

## Implementation

hoDCA is implemented in the julia language (0.6.2) [16], and depends directly on a) the ArgParse [17] module for command-line arguments and b) on the GaussDCA [18] module for performing preprocessing operations on the MSA and the implicit dependencies for those packages. A typical command-line call is julia hoDCA.jl Example.fasta Example.csv –No_A_Map=1 –Path_Map=A_Map.csv –MaxGap=0.9 –theta=0.2 –Pseudocount=4.0 –No_Threads=2 –Ign_Last=0 with input Example.fasta and output Example.csv. The latter consists of lists of all two-body contact scores *J*_*ij*_ separated by at least one residue along the backbone. The meaning of the remaining (optional) parameters will become clear in the following.

*General notes.* For inference of parameters {*h*_*i*_,*J*_*ij*_,*V*_*ijk*_}, we use the mean-field approximation as described in [15] with a reduced alphabet for three-body couplings. This is accomplished by a mapping

1 $$ \mu: \{l|l\leq q \} \to \left\{\alpha | \alpha \leq q_{\text{red}}\right\},  $$

with *q* being the full alphabet of the MSA and *q*_red_≤*q*. On the one hand, this accounts for the so called curse of dimensionality [19], occuring if the size of the MSA is too small to observe all possible *q*^3^ combinations for each *V*_*ijk*_. On the other hand, this significantly reduces memory usage and allows for a faster computation of contact prediction scores. The mapping *μ* can be specified by Path_Map, which is a csv file with every row representing a mapping. No_A_Map tells which row to choose. As the bottleneck is still the calculation of three-body couplings, it can be performed using parallel threads by specifying the No_Threads flag.

In traditional DCA, the last amino acid *q* usually represents the gap character and is not taken into account for score computation within the *l*_2_-norm. In hoDCA, each two-body coupling state *l*≤*q* contains contributions from {*n*≤*q*|*μ*(*n*)=*μ*(*l*)} due to the reduced alphabet. We therefore take gap contributions into account by default, which can be changed by the Ign_Last flag.

*MSA preprocessing.* The MSA is read in by the GaussDCA module, ignoring sequences with a higher amount of gaps than MaxGap, and subsequently converted into an array of integers. However, in contrast to GaussDCA, we check for the actual number of amino acids types contained in the MSA given. We, then, reduce the alphabet from *q*=21 to the number of present characters (amino acid types). Afterwards, the reweighting for every sequence $\vec {\sigma }^{(b)}$ is obtained by the GaussDCA module via $w_{b}=1/|\{a \in \{1,...,B \}: \text {difference}\left (\vec {\sigma }^{(a)},\vec {\sigma }^{(b)}\right) \leq \texttt {theta} \}|$, where the difference is computed by the percentage hamming distance [6]. The aim of reweighting is to reduce potential phylogenetic bias.

*Frequency computation.* Empirical frequency counts for the full alphabet are computed according to [6]

2 $$\begin{array}{*{20}l} \begin{aligned} & f_{i}(l) = \frac{1}{\lambda_{c}+B_{eff}} \left(\frac{\lambda_{c}}{q}+ \sum\limits_{b=1}^{B}w_{b} \cdot \delta\left(\sigma_{i}^{(b)},l\right) \right) \\ & f_{ij}(l,m) = \frac{1}{\lambda_{c}+B_{eff}} \left(\frac{\lambda_{c}} {q^{2}} \right.\\ &~~~~~~~~~~~~~~~~~~~~ \left.+\sum\limits_{b=1}^{B}w_{b} \cdot \delta\left(\sigma_{i}^{(b)},l\right) \delta\left(\sigma_{j}^{(b)},l\right) \right), \\ \end{aligned}  \end{array} $$

with *δ* being the Kronecker delta, *B* the number of sequences in the MSA, $B_{eff}={\sum \nolimits }_{b=1}^{B}w_{b}$ and *λ*_*c*_=Pseudocount·*B*_*eff*_. The Pseudocount parameter shifts empirical data towards a uniform distribution. This is necessary to ensure invertibility of the empirical covariance matrix in the mean-field approach.

Frequency counts for the reduced alphabet are computed via

3 $$\begin{array}{*{20}l} \begin{aligned} & f_{i}^{\text{red}}(\alpha) = \sum\limits_{\{ l | \mu(l)=\alpha \}} f_{i}(l) \\ & f_{ij}^{\text{red}}(\alpha,\beta) = \sum\limits_{\substack{\{ l | \mu(l)=\alpha \} \\ \{ m | \mu(m)=\beta \}}} f_{ij}(l,m) \\ & f_{ijk}^{\text{red}}(\alpha,\beta,\gamma) = \sum\limits_{\substack{\{ l | \mu(l)=\alpha \} \\ \{ m | \mu(m)=\beta \} \\ \{ n | \mu(n)=\gamma \}} } f_{ijk}(l,m,n). \\ \end{aligned} \end{array} $$

The computation of three-point frequencies takes some time and will be executed on No_Threads threads. For this, we parallelized their calculation over the sequence size *N*, meaning that the *i*-th process computes $f_{ijk}^{\text {red}}$ for all *k*≥*j*≥*i* and fixed *i*. Besides the parallelization scheme, three-point frequencies are preprocessed in the same manner as one- and two-point frequencies.

*Contact prediction scores.* Contact prediction scores follow directly from two-body couplings. Two-body couplings are obtained within the mean-field approximation by

4 $$\begin{array}{*{20}l} \begin{aligned} J_{ij}(l,m) \approx & -g_{ij}(l,m) \\ &+ \sum\limits_{\substack{k=1, \\ k \neq i,j}}^{N} \sum\limits_{n=1}^{q-1} g_{ijk}^{\text{red}}(\mu(l),\mu(m),\mu(n)) \cdot f_{k}(n),\\ \end{aligned} \end{array} $$

where *g*_*ij*_(*l*,*m*) is the inverse of the empirical two-point covariance matrix *e*_*ij*_(*l*,*m*)=*f*_*ij*_(*l*,*m*)−*f*_*i*_(*l*)*f*_*j*_(*m*). $g_{ijk}^{\text {red}}(\alpha,\beta,\gamma)$ is given by a relation to the three-point covariance matrix over the reduced alphabet

5 $$ \begin{aligned} e_{ijk}^{\text{red}}(\alpha,\beta,\gamma) = & f_{ijk}^{\text{red}}(\alpha,\beta,\gamma)+2f_{i}^{\text{red}}(\alpha)f_{j}^{\text{red}}(\beta)f_{k}^{\text{red}}(\gamma) \\ &-f_{ij}^{\text{red}}(\alpha,\beta)f_{k}^{\text{red}}(\gamma) \\ &-f_{ik}^{\text{red}}(\alpha,\gamma)f_{j}^{\text{red}}(\beta) \\ &-f_{jk}^{\text{red}}(\beta,\gamma)f_{i}^{\text{red}}(\alpha) \\ \end{aligned}  $$

via

6 $$ {{} \begin{aligned} g_{ijk}^{\text{red}}(\alpha,\beta,\gamma) = & \,-\,\sum\limits_{a_{1},b_{1},c_{1}=1}^{N} \sum\limits_{a_{2},b_{2},c_{2}\,=\,1}^{q_{\text{red}}-1} \left(e_{a_{1},b_{1},c_{1}}^{\text{red}}\left(a_{2},b_{2},c_{2}\right)\right. \\ &~~~~~~~~~~ \!\cdot \left.g_{i,a_{1}}^{\text{red}}\left(\alpha,a_{2}\right) \!\cdot\! g_{j,b_{1}}^{\text{red}}\left(\beta,b_{2}\right) \!\cdot\! g_{k,c_{1}}^{\text{red}}\left(\gamma,c_{2}\right) \right) \end{aligned}} \!\!,  $$

where *g*_*ij*_(*α*,*β*) is the inverse of the two-point covariance matrix over the reduced alphabet (see [15] for more details). For the calculation of scores, {*J*_*ij*_} are transformed into so called zero-sum gauge, satisfying ${\sum \nolimits }_{l}^{q} \hat {J}_{ij}(l,.)=\sum _{m}^{q} \hat {J}_{ij}(.,m)=0$, where "." stands for an arbitrary state via

7 $$ \begin{aligned} \hat{J}_{ij}(l,m)= & J_{ij}(l,m)+\frac{1}{q}\sum\limits_{r=1}^{q}\left[{\vphantom{\sum\limits_{s=1}^{q}}} -J_{ij}(r,m)-J_{ij}(l,r) \right.\\ &~~~~~+\left.\frac{1}{q}\sum\limits_{s=1}^{q}J_{ij}(r,s) \right] \\ &+\frac{1}{q_{\text{red}}}\sum\limits_{\substack{ k=1 \\ k \neq i,j}}^{N} \sum\limits_{\eta=1}^{q_{\text{red}}} \left[{\vphantom{\sum\limits_{s=1}^{q}}}V_{ijk}^{\text{red}}(\mu(l),\mu(m),\eta) \right.\\ & ~~~~~+\frac{1}{q}\sum\limits_{r=1}^{q} \left[{\vphantom{\sum\limits_{s=1}^{q}}} -V_{ijk}^{\text{red}}(\mu(r),m,\eta) \right.\\ & ~~~~~ -V_{ijk}^{\text{red}}(\mu(l),\mu(r),\eta) \\ &~~~~~ \left.\left.+\frac{1}{q}\sum\limits_{s=1}^{q}V_{ijk}^{\text{red}}(\mu(r),\mu(s),\eta) \right] \right] \\ \end{aligned}  $$

The purpose of the gauge transformation is to shift local bias from two-body couplings into local fields [8, 20]. Above calculations are the most time consuming parts and run on No_Threads threads. The final scores result from average product correction (APC) of *l*_2_ norm [21] via

8 $$ S_{ij}=\left\|\hat{\textbf{J}}_{ij} \right\|_{2} -\frac{\left\|\hat{\textbf{J}}_{:j} \right\|_{2}\left\|\hat{\textbf{J}}_{i:} \right\|_{2}}{\left\|\hat{\textbf{J}}_{::} \right\|_{2}}  $$

and $\left \| \hat {\textbf {J}}_{ij} \right \|_{2} = \sqrt {{\sum \nolimits }_{l,m=1}^{q} \hat {J}_{ij}(l,m)^{2}}$.

## Discussion

**Fig. 1:**
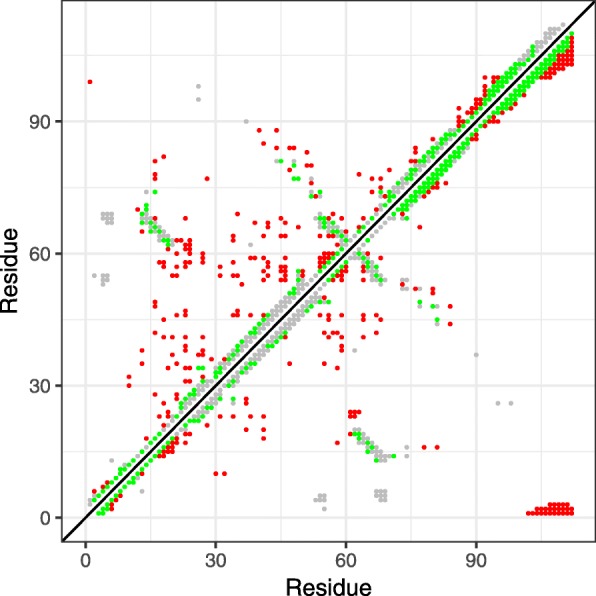
Contact map of pdb entry 1fx2A (gray) with true positives (green) and false positives (red) for a distance threshold of 7.5 Å. Upper left: classical mean-field DCA. Lower right: hoDCA with a mapping classification according to polarity [24]

A performance benchmark on the PSICOV-dataset [10], consisting of 150 proteins, is presented in [15]. For evaluating the performance of a single protein, the so called area under precision curve

9 $$ A:=\frac{1}{C}\sum\limits_{i=1}^{C}\frac{p_{i}}{i}  $$

was used, where *C* is the total amount of contacts and *p*_*i*_ is the number of true positives of the first *i* predictions. Figure 1 shows the predicted contact map of the protein data bank entry 1fx2A as an exemplary case. For this particular protein, the classical two-body DCA has an *A*-value of *A*≈0.5 while hoDCA shows a superior *A*≈0.72.

**Fig. 2:**
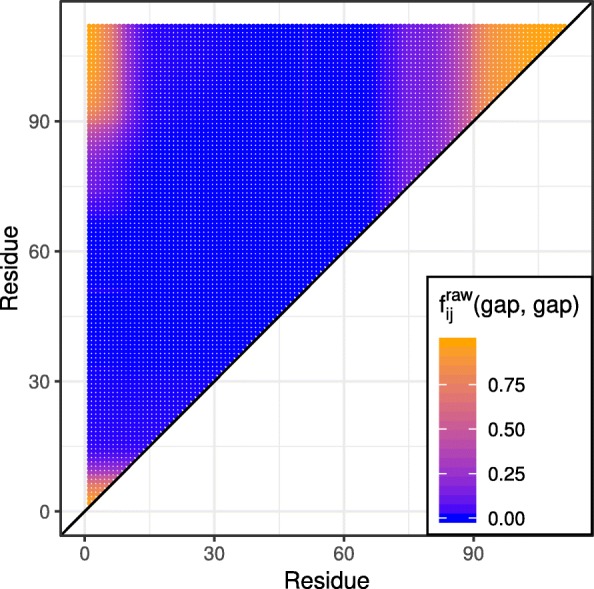
Raw gap-gap frequencies for pdb entry 1fx2A

Interestingly, the majority of hoDCA’s false positives are located in the lower and upper right corner of the contact map. We hypothesize that this finding is due to correlated gap regions in the corresponding MSA: For this particular pdb entry, many sequences were too short and had to be extended by gaps on both termini. This, in turn, leads to intra and inter correlations between the left and right termini. Figure 2 shows the two-point gap-gap frequencies of the non-preprocessed MSA (i.e. without sequence reweighting, pseudocount modification or deletion of sequences). As can be seen, there is indeed an accumulation of gap regions at the beginning and ending of the protein, thus possibly leading to false correlations.

## Results

**Fig. 3:**
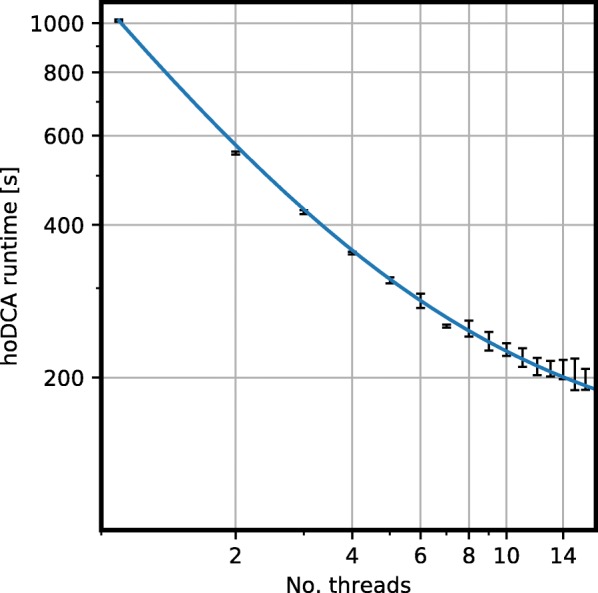
Runtime behaviour of hoDCA for PSICOV entry 1tqhA. The benchmark system was a Debian-operating server with two Intel(R) Xeon(R) CPU E5-2687W v2 @ 3.40GHz. Runtimes were taken for julia-compiled code, thus potential initalization overhead is omitted. The solid line shows a fit of Amdahl’s law

Figure 3 shows the runtime behavior of hoDCA when No_Threads are used for calculation of three-body terms. We used entry 1tqhA for the benchmark, which has one of the largest MSAs in the PSICOV dataset (*N*=242, *B*=18,170) and parameters as in Eq. (2). The overall speedup is about five-fold when executed on *n*≥12 threads in comparison to a single CPU core. A fit of Amdahl’s law *T*=*T*_0_·(1−*p*·(1−1/*n*)), with *T*_0_ being the single-threaded runtime and *n*=No_Threads, reveals the proportion of parallelized routines as *p*≈0.86. The serial runtime proportion of ≈0.14 comes mainly due to computation of two-body terms. Also note that we did not modify the standard julia parameters, meaning, e.g., a parallel computation of the matrix inverse by default.

## Conclusions

Higher-order interactions have been shown to have a strong influence on contact prediction in certain proteins [15, 22, 23]. Here, we implemented hoDCA, an extension of DCA by incorporating three-body couplings into the Hamiltonian. The accessible command-line user interface and the significant speedup within parallel execution make hoDCA suitable for contact prediction in a variety of proteins, using biochemical inspired alphabet reduction schemes. We hope to have made this method easily accessible for other researchers by this software release.

## Availability and requirements

**Project name:** hoDCA **Project home page:**http://www.cbs.tu-darmstadt.de/hoDCA/**Operating systems:** Linux, Windows, macOS **Programming language:** julia (0.6.2) **Other requirements:** julia packages Argparse, GaussDCA **License:** GNU General Public License v3, http://www.gnu.org/licenses/gpl-3.0.html**Any restrictions to use by non-academics:** Any commercial use is subject to a contractual agreement between involved parties.

## Abbreviations

APC: Average product correction
DCA: Direct-coupling analysis
MSA: Multiple sequence alignment
